# The phenotype‐driven computational analysis yields clinical diagnosis for patients with atypical manifestations of known intellectual disability syndromes

**DOI:** 10.1002/mgg3.1263

**Published:** 2020-04-26

**Authors:** Aleksandra Jezela‐Stanek, Elżbieta Ciara, Dorota Jurkiewicz, Marzena Kucharczyk, Maria Jędrzejowska, Krystyna H. Chrzanowska, Małgorzata Krajewska‐Walasek, Tomasz Żemojtel

**Affiliations:** ^1^ Department of Genetics and Clinical Immunology National Institute of Tuberculosis and Lung Diseases Warsaw Poland; ^2^ Department of Medical Genetics The Children’s Memorial Health Institute Warsaw Poland; ^3^ Mossakowski Medical Research Centre Neuromuscular Unit Polish Academy of Sciences Warsaw Poland; ^4^ Genomics Platform Berlin Institute of Health Berlin Germany; ^5^ Institute of Bioorganic Chemistry Polish Academy of Sciences Poznan Poland

**Keywords:** *AP4M1*, dysmorphology, *EP300*, HPO, intellectual disability patients, *KMT2A*, *NR2F1*, PhenIX, Phenomizer, *PURA*, *SATB2*, *SLC6A8*, *SMC1A*

## Abstract

**Background:**

Due to extensive clinical and genetic heterogeneity of intellectual disability (ID) syndromes, the process of diagnosis is very challenging even for expert clinicians. Despite recent advancements in molecular diagnostics methodologies, a significant fraction of ID patients remains without a clinical diagnosis.

**Methods, results, and conclusions:**

Here, in a prospective study on a cohort of 21 families (trios) with a child presenting with ID of unknown etiology, we executed phenotype‐driven bioinformatic analysis method, PhenIX, utilizing targeted next‐generation sequencing (NGS) data and Human Phenotype Ontology (HPO)‐encoded phenotype data. This approach resulted in clinical diagnosis for eight individuals presenting with atypical manifestations of Rubinstein–Taybi syndrome 2 (MIM 613684), Spastic Paraplegia 50 (MIM 612936), Wiedemann–Steiner syndrome (MIM 605130), Cornelia de Lange syndrome 2 (MIM 300590), Cerebral creatine deficiency syndrome 1 (MIM 300352), Glass Syndrome (MIM 612313), Mental retardation, autosomal dominant 31 (MIM 616158), and Bosch–Boonstra–Schaaf optic atrophy syndrome (MIM 615722).

## Graphical abstract

Due to extensive clinical and genetic heterogeneity of intellectual disability syndromes (ID), the process of diagnosis is very challenging even for expert clinicians. Despite recent advancements in molecular diagnostics methodologies, significant fraction of ID patients remains without clinical diagnosis. Here, in a prospective study on a cohort of 21 families (trios) with a child presenting with ID of unknown etiology, we executed phenotype‐driven bioinformatic analysis method, PhenIX, utilizing targeted next generation sequencing (NGS) data and Human Phenotype Ontology (HPO)‐encoded phenotype data. This approach resulted in clinical diagnosis for eight individuals presenting with atypical manifestations of syndromes with intellectual disability.

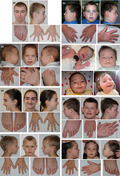

## 1 INTRODUCTION

Intellectual disability (ID) patients and their families often experience diagnostic odyssey. The process of diagnosis is very challenging even to expert clinicians, owing to the clinical and genetic heterogeneity of multiple ID disorders. The introduction of next‐generation sequencing (NGS) has revolutionized diagnostics for intellectual disability patients (Gilissen et al., 2014; Najmabadi et al., 2011). Another significant recent methodological advancement has been the implementation of Human Phenotype Ontology (HPO) (Kohler et al., 2017), providing standardized vocabulary allowing for the description of patient phenotypes. In turn, bioinformatic tools such as Exomiser, PhenIX, Genomiser (Smedley et al., 2015, 2016; Zemojtel et al., 2014) have been implemented, which use the power of ontology‐based searches enabling comparison of the patient's phenotype with known disease phenotypes (Smedley et al., 2015).

The role of de novo mutations in families with sporadic ID cases has been implied (Robinson, Krawitz, & Mundlos, 2011; Veltman & Brunner, 2012). The reported prevalence of autosomal de novo mutations causing disease in patients with ID ranges from 16% to 60%, and for autosomal recessive intellectual disability patients is reported to be 10% (Gilissen et al., 2014; Jamra, 2018).

In this prospective study on a cohort of 21 families (trios) with a child presenting with intellectual disability, we have implemented into our diagnostic clinical workflow the bioinformatic tool, PhenIX (Zemojtel et al., 2014), that utilizes next‐generation sequencing and HPO‐encoded phenotype data, which allowed us to provide a clinical diagnosis for 8 ID patients.

## 2 MATERIALS AND METHODS

Patients included in this study had been well‐characterized clinically, and represented sporadic, familial cases, and had been classified as “Nonspecific intellectual disability patients.” Patient phenotypes were encoded using HPO annotations (Table 1; Table S1).

**Table 1:** Genotypes and phenotypes of the patients diagnosed in the study. Only the most striking phenotypic features are listed. Table S1 contains the full phenotypic spectrum of the diagnosed patients as well as reported phenotypic spectrums associated with the diagnosed syndromes

Patient ID; Age; Gender; Diagnosis	Phenotypic Features (HPO terms)	Genotype^a^; Pathogenicity^b^; Segregation
Patient 1; 14y; m; Rubinstein–Taybi syndrome 2, RSTS2; AD (MIM 613684)	Intellectual disability, moderate (HP:0002342), Facial grimacing (HP:00002273), Growth delay (HP:0001510), Bilateral cryptorchidism (HP:0008689), Microcephaly (HP:0000252), Narrow palpebral fissures (HP:0000581), Dental crowding (HP:0000678),), High, narrow palate (HP:0002705), Abnormality of the fingertips (Square)(HP:0001211)	*EP300*,NM_001429.3: c.[5783dup];[=], p.(Met1928Ilefs*145), novel;*probably pathogenic*, MAF = 0; de novo
Patient 2; 8.5y, m; Spastic Paraplegia 50, SPG50; AR (MIM 612936)	Intellectual disability, severe (HP:0010864), Seizures (HP:0001250), Generalized hypotonia (HP:0001290), Microcephaly (HP:0000252), Tapered fingers (HP:0001182), Abnormal myelination (HP:0012447)	*AP4M1*, NM_004722.2: c.[566del];[916C > T]; p.(Leu189Trpfs*10)/ p.(Arg306*); novel/known (ClinVar:RCV000680158.1); *pathogenic/probably pathogenic* MAF = 0/ MAF = 0.000039 (GnomAD); *paternal/maternal*
Patient 3; 4y; f; Wiedemann–Steiner syndrome, WDSTS; AD (MIM 605130)	Intellectual disability, moderate (HP:0002342), Failure to thrive (HP:0001508), Muscular hypotonia (HP:0001252)/Generalized hypotonia (HP:0001290), Localized hirsutism (HP:0009889), Thin upper lip (HP:0000219), Clinodactyly of the fifth fingers (HP:0004209), Tapered fingers (HP:0001182)	*KMT2A*, NM_001197104.1: c.[4012 + 2T>A];[=]; p.?;novel; probably pathogenic (the substitution is located in the donor splice site,MaxEnt: −100.0%, NNSPLICE: −100.0%, SSF: −100.0%), MAF = 0; de novo
Patient 4; 5y; f;Cornelia de Lange syndrome 2, CDLS2; XLD (MIM 300590)	Intellectual disability, severe (HP:0010864), Growth delay (HP:0001510), Seizures (HP:0001250)/EEG abnormality (HP:0002353), Hyperactivity (HP:0000752), Hirsutism (HP:0001007), Microcephaly (HP:0000252), Arched eyebrows (HP:0002553), Anteverted nares (HP:0000463)	*SMC1A*, NM_006306.3: c.[238G > T];[=], p.(Val80Phe); novel; *probably pathogenic* (SIFT: Deleterious, MutationTaster: disease causing), MAF = 0; de novo
Patient 5; 14y; f; Glass syndrome; AD (OMIM 612313)	Intellectual disability, severe (HP:0010864), Marfanoid habitus (HP:0001519), Scoliosis (HP:0002650), Synophrys (HP:0000664), Long fingers (HP:0100807)	*SATB2*: NM_015265.3:c.[716del];[=], p.(Arg239Glnfs*20), novel; *probably pathogenic*; de novo
Patient 6; 10y; m; Bosch–Boonstra–Schaaf optic atrophy syndrome, BBSOAS; AD (MIM615722)	Intellectual disability, severe (HP:0010864), Cerebral palsy (HP:0100021), Seizures (HP:0001250), Gait imbalance (HP:0002141), Recurrent infections (HP:0002719), Narrow hands (HP:0004283), Narrow foot (HP:0001786)	*NR2F1,* NM_005654.5: c.[1217T > C];[=]; p.(Met406Thr); known (ClinVar: RCV000477887.1); *probably pathogenic* (MutationTaster: disease causing), MAF = 0; de novo
Patient 7; 7.5y; m; Cerebral creatine deficiency syndrome 1, SLC6A8; XLR (MIM300352)	Intellectual disability, moderate (HP:0002342), Autism (HP:0000717), Increased muscle tone (HP:0001276), Velvety skin (HP:0000977), Hyperextensibility of the finger joints (HP:0001187)	*SLC6A8,* NM_005629.3: c.[224T > C];[=];p.(Val75Ala);missense, novel;*probably pathogenic;* de novo
Patient 8; 4y; f; Mental retardation, autosomal dominant 31, MRD31; AD (MIM 616,158)	Intellectual disability (HP:0001249), Self‐injurious behavior (HP:0100716), Obesity (HP:0001513), Muscular hypotonia (HP:0001252), Loose skin (HP:0000973)	*PURA,* NM_005859.4: c.[3G > A];[=];p.?; start loss, novel; *probably pathogenic* (SIFT: Deleterious, MutationTaster: disease causing), MAF = 0; de novo

^a^ The nomenclature of molecular variants follows the Human Genome Variation Society guidelines (HGVS, http://varnomen.hgvs.org/) using human cDNA sequences from RefSeq database.

^b^ Molecular variants were assessed by pathogenicity prediction tools: SIFT and MutationTaster software for nucleotide changes localized in coding sequence, and MaxEnt, NNSPLICE or SSF for nucleotide changes identified in intronic sequence. ClinVar database was search for known pathogenic variants (https://www.ncbi.nlm.nih.gov/clinvar/). The minor allele frequency (MAF) as recorded in ExAC and GnomAD databases.

Genomic DNA was extracted by an automated method (MagnaPure, Roche) from peripheral blood samples of the patients and their parents. Next‐generation sequencing of the disease‐associated genome targeting ~2,800 genes known to be associated with Mendelian disorders (DAG; disease‐associated genome) was performed for all patients (patients 1–8; Zemojtel et al., 2014). In three cases (patients 6–8) undiagnosed by using DAG gene panel, whole‐exome sequencing (WES) was further conducted. Prior to the library preparation, DNA samples were quantified using Qubit dsDNA HS Assay Kit (Life Technologies), and DNA degradation status was estimated by 1% agarose gel electrophoresis. About 50 ng of high‐quality genomic DNA was used for library construction with SureSelect^XT^ library prep kit (Agilent) for DAG Panel sequencing and SureSelect Human All Exon V6 (Agilent) for WES. Each library was qualified using Bioanalyzer (Agilent) and quantified using Qubit (Life Technologies). Before the sequencing run, cluster amplification was carried out on cBot (Illumina) with TruSeq PE Cluster Kit v4 (Illumina). HiSeq4000 (Illumina) paired‐end sequencing (2 × 100 bp) was performed using TruSeq SBS Kit v4 (Illumina) with the required mean sequence coverage × 140 (GE20 ≥ 98%).

NGS data were analyzed using an in‐house procedure, described in detail previously (Zemojtel et al., 2014). To detect potentially causative variants from DAG and whole exome data, we applied a bioinformatic tool PhenIX (Phenotypic Interpretation of eXomes) (Zemojtel et al., 2014).

For each variant PhenIX computes a pathogenicity score and a phenotype score measuring similarity between encoded by HPO terms variant‐associated disease phenotypes and the patient's phenotype. Finally, by using both scores the software ranks detected gene variants.

The candidate pathogenic variants were verified in the probands and parents by Sanger sequencing using BigDye Chemistry (Applied Biosystems).

The nomenclature of molecular variants follows the Human Genome Variation Society guidelines (HGVS, http://varnomen.hgvs.org/). The human cDNA sequence of the appropriate gene is according to the Genebank (https://www.ncbi.nlm.nih.gov/genbank/).

## 3 RESULTS

By applying PhenIX bioinformatic method to NGS and phenotypic data of 21 examined ID patients, the etiological diagnosis was made in eight cases. The following eight syndromes were diagnosed: Rubinstein–Taybi syndrome 2 (MIM 613684), Spastic Paraplegia 50 (MIM 612936), Wiedemann–Steiner syndrome (MIM 605130), Cornelia de Lange syndrome 2 (MIM 300590), Cerebral creatine deficiency syndrome 1 (MIM 300352), Glass Syndrome (MIM 612313), Mental retardation, autosomal dominant 31 (MIM 616158), and Bosch–Boonstra–Schaaf optic atrophy syndrome (MIM 615722).

All 21 ID patients, before enrolment in the study, underwent detailed clinical and phenotypical examination and were classified as “Nonspecific intellectual disability” patients. In Table 1, we list the most striking of their phenotypical features.

**Figure 1:**
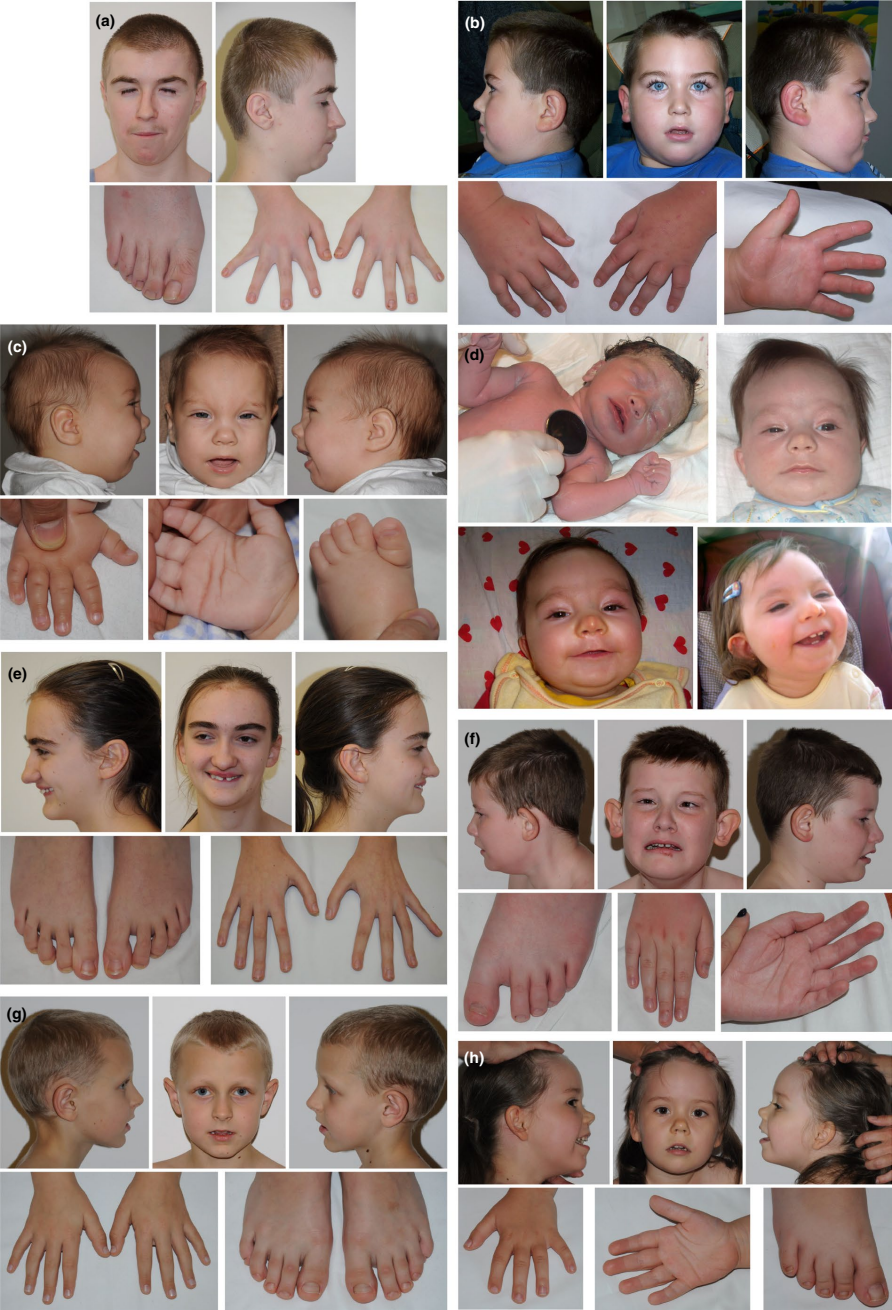
Photos of the patients diagnosed in this study. (a) Rubinstein–Taybi syndrome 2 (MIM 613684) patient. (b) Spastic Paraplegia 50 (MIM 612936) patient. (c) Wiedemann–Steiner syndrome (MIM 605130) patient. (d) Cornelia de Lange syndrome 2 (MIM 300590) patient. (e) Glass Syndrome (MIM 612313) patient. (f) Bosch–Boonstra–Schaaf optic atrophy syndrome (MIM615722) patient. (g) Cerebral creatine deficiency syndrome 1 (MIM 300352) patient. (h) Mental retardation, autosomal dominant 31 (MIM 616158) patient

Patient 1, the boy diagnosed with Rubinstein–Taybi syndrome (Figure 1a), was born at term with weight 1,880 g (< 3c), length 48 cm (25‐50c), OFC 28 cm (<3c), and chest circumference 28 cm. Subsequently, bilateral cryptorchidism and supernumerary nipples were noted. His development was delayed, with the acquisition of the sitting position at 12 months, of the walk at 2 years and the speech at 4 years. Moreover, growth deficiency with delayed carpal bone ossification (bone age 10 years for chronological age 12.6 years) was diagnosed. Psychological consultation revealed a moderate degree of ID (IQ 50). At the time of diagnosis (14 years), he presented with some distinctive facial appearance, marked by very short palpebral fissures. After clinical verification of molecular result, we defined it as facial grimacing, resulting from the closing of the eyes during the laughing (Figure 1a). His fingers were somewhat squared at tips, and thumbs only slightly broader. He was outgoing with cheerful mood, could not read, and attended the special school.

Patient 2 (Figure 1b), diagnosed with Spastic Paraplegia 50, was referred for genetic counseling because of developmental delay, hypotonia, and epilepsy. Moreover, he presented with microcephaly, obesity, pes equinovarus (also noted in his mother), had a small penis, and—when aged 8.5 years—could not speak (used the finger to express his needs). During our follow‐up, at the age of 12, increased muscle tone was first noted. The boy was born in 38th week of gestation with a weight of 3,420 g (50‐75c), and OFC 33.5 cm (50c). His anthropometric parameters changed with age as follows: at the age of 4.5 years—weigh 31 kg (>97c), height 115 cm (90c), while the age of 8.5 years—weigh 57 kg (>97c), height 140 cm (90c), OFC 50 cm (<3c). He was very calm, understood many commands, and presented with laughter attacks and occasional sleep disturbances.

Patient 3, the girl diagnosed with Wiedemann–Steiner syndrome (Figure 1c) was born at term with normal parameters—weigh 3,550 g (75‐90c), length 54 cm (75‐90c), OFC 34 cm (50‐75c), chest circumference 34 cm). Neonatal period was complicated by hyperbilirubinemia (15.9 mg%), laryngeal stridor, and muscular hypotonia. Then poor weight gain was noted. At 1 year of age secondary microcephaly occurred (OFC 42.5 cm). She sat unsupported at 1.5 year, started walking from 3 years 2 months. At the age of diagnosis (4 years), the proband could not speak, had moderate ID.

Patient 4, the girl diagnosed with Cornelia de Lange syndrome 2 (Figure 1d) was referred for genetic testing because of developmental delay, epilepsy (partial seizures), growth delay, and heart defect (ASDII, PDA). Epilepsy occurred during the neonatal period and was difficult to control. MRI performed at the age of 2 years showed hypoplasia of frontal part of cerebral falx (also noted at 1 year) and frontal lobes asymmetry (P > L). Moreover, hyperactivity with poor attention span and no visual contact were observed. Her facial phenotype was not specific. The reliable dysmorphological assessment was, nevertheless, difficult because of the lipoma on the nose.

Patient 5 (Figure 1e) diagnosed with Glass Syndrome was born as a second child of healthy but consanguineous parents (mother's and father's grandfathers were brothers). Her psychomotor development was delayed. She started walking independently at 2 years, vocalized first words at 1.5–2 years. At school age, the patient was diagnosed with severe ID (IQ 35). At the age of 14 years, she attended a special school, presented poor manual ability and no writing nor reading skills. Her body habitus was thin and marfanoid, with long fingers and delicate dermatoglyphics.

Moreover, scoliosis and gait imbalance were noted. The proband's craniofacial phenotype was marked by posteriorly rotated ears, synophrys, diastema and crowded teeth, short philtrum, long and prominent nose. She had a friendly and happy personality.

Patient 6, the boy diagnosed with Bosch–Boonstra–Schaaf optic atrophy syndrome (Figure 1f) was referred to genetic counseling with cerebral palsy, epilepsy, and optic anomalies (nystagmus horizontalis, amblyopia). Epilepsy was noted from his 4th month of age, diagnostics MRI revealed delayed myelination, and optic nerve dysplasia was suspected. He suffered from recurrent infections. At the age of 7 years, the patient could not speak, presented with wide‐based gait and was diagnosed with severe ID. No specific dysmorphism was noted; however, his phenotype was marked by high forehead, large and protruding ears, narrow hands with long fingers and dorsal dimpling (like in patients with Cohen syndrome), sandal gap, long toe, pes planovalgus, and upper and lower limbs contractures.

Patient 7 (Figure 1g) diagnosed with cerebral creatine deficiency syndrome, did not present with any dysmorphic features, however, had velvet skin, flat feet and hyperextensible finger joints. In infancy, physiotherapy was conducted because of increased muscle tone. The boy started to walk at the age of 1.5 years but presented “toe‐walking.” In differential diagnosis, fragile X syndrome was excluded. Brain imaging (MRI) showed normal result. Based on psychological tests, his intellectual disability was classified as moderate with an autism spectrum disorder.

Patient 8 (Figure 1h), diagnosed with Mental retardation, autosomal dominant 31, was initially been suspected of suffering from metabolic disease. She was born after second pregnancy complicated by maternal genital tract infection and weak fetal movements. In the perinatal period hypotonia, somnolence, apathy, poor sucking and loss of weight during the first months were noted. She presented poor reaction for sounds and poor interest in surroundings as well. Based on these symptoms, molecular tests for inherited metabolic diseases and Prader–Willi syndrome were conducted, giving normal results. At the age of 1 year, the genetic counseling showed: severe hypotonia (no crawling and turning around), overweight, temporal narrowing, broad face, almond‐shaped eyes, slightly up‐slanted palpebral fissures, hypertelorism, dark‐blond hair, flat nasal base with upturned tip, downturned mouth corners, puffy hands and feet, deep dermatoglyphics, and hypoplastic external female genitals. Then, follow‐up at 4 years revealed: proportionate stature, broad‐based walking, aggression, and auto aggression, increased sensitivity to touch, hyperextensible and loose skin, hypomimic face, epicanthus, long philtrum, triangular mouth shape, thin upper lip, thick helix and prominent earlobe ("red blood corpuscles" shaped), soft hands, minimal V clinodactyly, pes planovalgus, and knee hyperextension.

## 4 DISCUSSION

During the last decades, the clinical model of dysmorphic syndrome recognition has changed diametrically. Before the introduction of microarray chromosomal analyses or, the more, next‐generation sequencing, molecular tests were ordered based on clinicians’ suspicion from clinical examination and patients’ history (“phenotype‐first” approach). We have currently entered the era of reverse dysmorphology, where “genotype‐first” model of genetic assessment is favored (Douzgou et al., 2014; Miller et al., 2010). Still, detailed phenotypical evaluation of patients is critical for the process of diagnosis.

We performed dysmorphological examinations before and after the clinical diagnosis was established. We have encoded patient phenotypes using the Human Phenotype Ontology (HPO) terms (Table 1; Table S1), which provide a standardized vocabulary of phenotypic abnormalities encountered in human disease (Kohler et al., 2017). To identify causative gene variants, we have utilized the bioinformatic method called Phenotypic Interpretation of eXomes (PhenIX) (Zemojtel et al., 2014). It evaluates and ranks variants based on pathogenicity and semantic similarity of patients’ phenotypes described by Human Phenotype Ontology (HPO) terms to those of known Mendelian diseases. In computer simulations, ranking genes based on the variant score put the true gene in first place <5% of the time, while PhenIX placed the correct gene in first place more than 86% of the time (Zemojtel et al., 2014). Unlike other programs, such as Exomiser, which allow for discovering new disease genes via model organism data, PhenIX was developed for supporting clinical diagnostics of patients that were subjects to diagnostic odyssey (i.e., patients that cannot be diagnosed).

By utilizing the diagnostic workflow based on PhenIX, we established the diagnosis for 8 of 21 patients (38%, Table 1) that were subjects to diagnostics odyssey. Our clinical reassessment of these patients let us to following conclusions.

The boy (Patient 1) with Rubinstein–Taybi syndrome (RTST2) had no classical features, which are broad toes/thumbs or long columella extending below the alae nasi. Besides, narrow and short palpebral fissures distorted his facial features. Because of somewhat broad tips of his fingers and downwards position of the palpebral fissures in differential dysmorphological diagnostics, we did consider Rubinstein–Taybi syndrome, and analysis of the *CREBBP* was performed, revealing no abnormality. At that time, mutations in the *EP300* were reported very rarely. Therefore, we decided not to conduct further tests toward RSTS. According to recent study, patients with RSTS2 may present less severe facial phenotype than RSTS patients with *CREBBP* mutations, and in some cases lack of the characteristic hands and feet malformations is noted (Fergelot et al., 2016; Solomon et al., 2015). Of note, RSTS2 and RSTS patients have been reported to show facial grimacing provoked by the closing of the eyes during the laughing. The patient described by us was, however, characterized by a significant shortening of palpebral fissures, which notably affected his facial appearance. Besides, mentioned grimacing was not always observed, but only during the patient's laughter. These features certainly influenced our clinical assessment and distanced us from the diagnosis of Rubinstein–Taybi syndrome before the molecular examination.

During our observation, the facial phenotype of the girl diagnosed with Wiedemann–Steiner syndrome (WSS), Patient 3, has been notably changing and become more specific for WSS, which is in line with the literature (Baer et al., 2018). Another of the patient's particular features was hypertrichosis cubiti, which was reported in 61% of recently reviewed WSS cases (Baer et al., 2018). In the infancy or early childhood, such hypertrichosis was observed by us. It became apparent as she got older, and then—as we found in medical records—turned our attention.

The gestalt of the Patient 4, the girl diagnosed with Cornelia de Lange syndrome 2 (CDLS2) was definitely mild, as compared to classical phenotype of *NIPBL* patients, which is consistent with reported CDLS type 2 cases. Her anthropometric parameters did not improve with age, and no fingers/limbs anomalies were noted. The girl's features including somewhat arched and pencilled eyebrows, short and upturned nose together with downturned lips were noted only in the neonatal period and characterized as very subtle. During further observation, this facial appearance has changed, that was partially resulting from nasal lipoma.

The diagnosis of Glass syndrome (GLASS, MIM 612313), in Patient 5, was not taken into consideration as there were only a few cases with heterozygous mutations in the *SATB2* reported at the time of the patient's examination. One feature frequently published for Glass syndrome patients is hand cleft palate, which was not noted in the patient (Docker et al., 2014; Leoyklang et al., 2007; Rosenfeld et al., 2009).

The phenotype of the patient (Patient 6) with Bosch–Boonstra–Schaaf optic atrophy syndrome (BBSOAS) was not specific enough to allow for diagnosis based on dysmorphic features. Analysis of medical records, after the molecular result, let to clinical verification and confirmation of the disorder.

The diagnosis of Spastic Paraplegia 50 (SPG50, MIM 612936), Cerebral creatine deficiency syndrome 1 (MIM 300352, CCDS1), as well as Mental retardation, autosomal dominant 31 (MIM 616158, MRD31), would not be possible without the application of next‐generation sequencing. None of these diseases is manifesting with specific dysmorphic features. The case of the Patient 2, diagnosed with SPG50, was even complicated by the presence of pes equinovarus also in his mother, as well as perinatal complications (amniotic fluid aspiration, intrauterine hypoxia, hyperbilirubinemia at 15 mg%).

## 5 CONCLUSIONS

Based on the current state‐of‐the‐art, and in line with the data presented in this paper, de novo mutations constitute the most important cause of intellectual disability.

Given the authors’ long‐standing experience in clinical dysmorphology, and the ability to recognize many of classical dysmorphic syndromes, it is still very challenging for them to recognize their mild/unusual presentations. In the absence of recognized phenotypic features specific for a particular monogenic syndrome, the application of unbiased genome‐wide next‐generation sequencing is mandatory to facilitate clinical diagnosis.

A significant milestone in the clinical diagnostics of genetic disease was the implementation of recently developed bioinformatics tools like PhenIX (Zemojtel et al., 2014), that utilize next‐generation sequencing and Human Phenotype Ontology‐encoded phenotypic data and employ ontology‐based searches to match patients’ phenotypes against the known disease phenotypes. It should be, however, emphasized that the performance of these tools is heavily dependent on the quality of the phenotypic examination of patients by expert dysmorphologists. These methodologies can be further complemented by frontal image analysis by deep‐learning algorithms allowing to quantify the phenotypic similarity (Hsieh et al., 2019).

## CONFLICT OF INTEREST

The authors declare no conflict of interest.

## AUTHOR CONTRIBUTIONS

E.C., D.J., M.K., and T.Z, implemented molecular diagnostics, A.J.‐S., M.K.‐W., and T.Z. designed the study, A. J.‐S., M.K.‐W., and T.Z. analyzed the data, K.H.C., C.E., D.J., A.J.‐S., M. K.W., and T.Z. wrote the manuscript, M.K.‐W., J.M., and K.H.C. examined patients.

## Supporting information

Table S1
