# Supplementary material for: The phenotype‐driven computational analysis yields clinical diagnosis for patients with atypical manifestations of known intellectual disability syndromes
Source: Mol Genet Genomic Med. 2020 Apr 26;8(9):e1263. doi: 10.1002/mgg3.1263 (PMC7507388; doi:10.1002/mgg3.1263)
Supplement: Supplementary file 1 — Table S1 [file MGG3-8-e1263-s001.docx]

Supplementary Table 1. Genotypes and full phenotypic features of the patients diagnosed in the study.

| **Patient ID; Age; Gender; Diagnosis** | **Phenotypic Features (HPO terms)** | **Genotype*;**  **Pathogenicity**; Segregation** | **Recorded phenotypic spectrums prior to this study** |
| --- | --- | --- | --- |
| Patient 1;  14y; m;  Rubinstein-Taybi syndrome 2, RSTS2; AD  (MIM 613684) | Intellectual disability, moderate (HP:0002342), Facial grimacing (HP:00002273), Growth delay (HP:0001510), Bilateral cryptorchidism (HP:0008689), Microcephaly (HP:0000252), Narrow palpebral fissures (HP:0000581), Dental crowding (HP:0000678), ), High, narrow palate (HP:0002705), Abnormality of the fingertips (Square)(HP:0001211), Intrauterine growth retardation (HP:0001511), Dental malocclusion (HP:0000689), Precoccious puberty (HP:0000826), Localized hirsutism (HP:0009889), Flat occiput (HP:0005469), Abnormal hair pattern HP:0010720), Posteriorly rotated ears (HP:0000358), Broad eyebrows (HP:0011229), Epicanthus (HP:0000286), Long eyelashes (HP:0000527), Narrow nose (HP:0000460), Prominent (pointed) nasal tip (HP:0005274), Long philtrum (HP:0000343), Carious teeth (HP:0000670 Supernumerary nipples (HP:0002558), Long fingers (HP:0100807), Broad thumbs (slightly) (HP:0011304), Genu valgum (HP:0002857), Hallux valgus (HP:0001822), Flat feet (HP:0001763), Valgus foot deformity (HP:0080810), Abnormality of carpal bone ossification (HP:0006257) | *EP300*, NM_001429.3: c.[5783dup];[=], p.(Met1928Ilefs*145), novel; *probably pathogenic*, MAF=0;  *de novo* | Varying degrees of developmental delay, in some cases lack of the classic feet and hands malformations, less severe facial phenotype than patients with *CREBBP* mutations^1^ |
| Patient 2 8.5y, m;  Spastic Paraplegia 50, SPG50; AR  (MIM 612936) | Intellectual disability, severe (HP:0010864), Seizures (HP:0001250), Generalized hypotonia (HP:0001290), Microcephaly (HP:0000252), Tapered fingers (HP:0001182), Abnormal myelination (HP:0012447), Absent speech (HP:0001344), Inappropriate laughter (HP:0000748), Obesity (HP:0001513), Recurrent infections (HP:0002719), Sleep disturbance (HP:0002360), Abnormality of amniotic fluid, (HP:0001560) Hyperbilirubinemia (HP:00002904), Abnormal hair pattern (HP:0010720), High palate (HP:0000218), Protruding tongue (HP:0010808), Abnormality of the Achilles tendon (HP:0005109), Equinovqrus deformity (HP:0008110), Defect in the atrial septum (HP:0001631), Spacticity (from 10y) (HP: 0001257) | *AP4M1*, NM_004722.2: c.[566del];[916C>T]; p.(Leu189Trpfs*10) / p.(Arg306*);  novel/known  (ClinVar: RCV000680158.1);  *pathogenic/probably pathogenic*  MAF=0 / MAF =0.000039 (GnomAD);  *paternal/maternal* | Severe intellectual disability, severe speech disorder, microcephaly, seizures, hypotonia (neonatal period), hypertonia, spasticity, Babinski sign, bitemporal narrowing, broad nasal bridge, bulbous nose, short philtrum, ventriculomegaly thin corpus callosum^2,3^ |
| Patient 3; 4y; f;  Wiedemann-Steiner syndrome, WDSTS; AD  (MIM 605130) | Intellectual disability, moderate (HP:0002342), Failure to thrive (HP:0001508), Muscular hypotonia (HP:0001252)/Generalized hypotonia (HP:0001290), Localized hirsuitism (HP:0009889), Thin upper lip (HP:0000219), Clinodactyly of the 5th fingers (HP:0004209), Tapered fingers (HP:0001182), Decreased body weight (HP:0004325), Absent speech (HP:0001344), Finger joint hyperextensibility (HP:0006158), Joint hypermobility (HP:0001382), Increased nuchal translucency (HP:0010880), Breech presentation (HP:0001623), Laryngeal stridor (HP:0006511), Gastroesophageal reflux (HP:0002020), Delayed eruption of primary teeth (HP:0000680), Microcephaly (HP:0000252), Hypertelorism (HP:0000316), Epicanthus (HP:0000286), Wide nose (HP:0000445), Anteverted nares (HP:0000463), Retrognathia (HP:0000278), Open mouth (HP:0000194), High palate (HP:0000218), Hypoplasia of the dental enamel (HP:0006297), Carious teeth (HP:0000670), 2-3 finger syndactyly (slight) (HP:0001233), Single tranverse palmar crease (HP:0000954), Deep palmar creases (HP:0006191), Abnormality of the fingernails (small) (HP:0001231), Abnormality of the distal phalanx of finger (HP:0009832), Short fingers (HP:0009803), Sandal gap (HP:0001852), Abnormality of the hallux (HP:0001844) | *KMT2A*, NM_001197104.1:  c.[4012+2T>A];[=]; p.?;  novel; probably pathogenic (the substitution is located in the donor splice site, MaxEnt: -100.0%, NNSPLICE: -100.0%, SSF: -100.0%), MAF=0;  *de novo* | Intellectual disability, short stature, hypertrichosis (especially cubiti), strabismus, hypotonia, flat face, thick eyebrows/synophrys,  hypertelorism/telecanthus,  vertically narrow, palpebral fissures, high nasal bridge, broad nose and nasal tip, long philtrum, thin upper lip, dysmorphic ears, some patients display some level of clinical overlap with Kabuki syndrome and Cornelia de Lange syndrome^4^ |
| Patient 4; 5y; f;  Cornelia de Lange syndrome 2, CDLS2; XLD  (MIM 300590) | Intellectual disability, severe (HP:0010864), Growth delay (HP:0001510), Seizures (HP:0001250)/EEG abnormality (HP:0002353), Hyperactivity (HP:0000752), Hirsutism (HP:0001007), Microcephaly (HP:0000252), Arched eyebrows (HP:0002553), Anteverted nares (HP:0000463), Short attention span (HP:0000736), Myopia (HP:0000545), Stridor (HP:0010307), Cavernous hemangioma (HP:0001048), Low posterior hairline (HP:0002162), Low-set, posteriorly rotated ears (HP:0000368), Epicanthus (HP:0000286), Abnormality of the nasal bridge (HP:0000422), Abnormality of finger (long) (HP:0001167), Tapered fingers (HP:0001182), 3-methylglutaconic aciduria (HP:0003535), Elevated hapatic transaminases (HP:0002910), Hypercholesterolemia (HP:0003124), Flat feet (HP:0001763)/Pes planus (HP:0001763), Defect in the atrial septum (HP:0001631), Patent ductus arteriosus (HP:0001643), Abnormality of the myocardium (HP:0001637), Abnormality of the falx cerebri (HP:0010653) | *SMC1A*, NM_006306.3: c.[238G>T];[=], p.(Val80Phe); novel;  *probably pathogenic* (SIFT: Deleterious, MutationTaster: disease causing), MAF=0;  *de novo* | Tendency to postnatal weight normalization, females typically have a milder phenotype compared to those with *NIPBL* mutations, particularly with respect to limb reduction defects, facial phenotypes somewhat less discriminative than those associated with *NIPBL^5^* |
| Patient 5; 14y; f;  Glass syndrome;  AD (OMIM 612313) | Intellectual disability, severe (HP:0010864), Gait imbalance (HP:0002141), Marfanoid habitus (HP:0001519), Scoliosis (HP:0002650), Synophrys (HP:0000664), Long fingers (HP:0100807), Posteriorly rotated ears (HP:0000358), Prominent nose (HP:0000448), Short philtrum (HP:0000322), Misalignment of teeth (HP:0000692), Widely spaced teeth (HP:0000687), Abnormal dermatoglyphics (HP:0007477) | *SATB2*:  NM_015265.3: c.[716del];[=], p.(Arg239Glnfs*20), novel;  *probably pathogenic*;  *de novo* | Developmental delay/ intellectual disability, limited/absent speech, behavioral problems, hypertelorism, downslanted palpebral fissures, micrognathia, cleft palate, crowded teeth, macrodontia, osteopenia, osteoporosis^6^ |
| Patient 6; 10y; m;  Bosch-Boonstra-Schaaf optic atrophy syndrome, BBSOAS; AD  (MIM615722) | Intellectual disability, severe (HP:0010864), Cerebral palsy (HP:0100021), Seizures (HP:0001250), Gait imbalance (HP:0002141), Recurrent infections (HP:0002719), Narrow hands (HP:0004283), Narrow foot (HP:0001786), Absent speech (HP:0001344), Short attention span (HP:0000736), Broad-based gait (HP:0002136), Abnormality of pain sensation (HP:0010832), Amblyopia (HP:0000646), Horizontal nystagmus (HP;0000666), Visual impairment (HP:0000505), Limb joint contracture (HP:0003121), High forehead (HP:0000348), Protruding ear (HP:0000411), Widely spaced teeth (HP:0000687), Abnormality of the distal phalanx of the thumb (HP:0009617), Long fingers (HP:0100807), Sandal gap (HP:0001852), Valgus foot deformity (HP:0080810), Flat feet (HP:0001763), Delayed myelination (HP:0012448) | *NR2F1,* NM_005654.5: c.[1217T>C];[=]; p.(Met406Thr);  known (ClinVar: RCV000477887.1);  *probably pathogenic*  (MutationTaster: disease causing), MAF=0;  *de novo* | Intellectual disability, optic atrophy, cerebral visual impairment, variable dysmorphic features, patients with variants in the ligand-binding domains were reported to have a milder degree of developmental delay, no hypotonia, no speech defects, no seizures, or no repetitive behaviors^7^ |
| Patient 7; 7.5y; m;  Cerebral creatine deficiency syndrome 1,  SLC6A8; XLR  (MIM 300352) | Intellectual disability, moderate (HP:0002342), Autism (HP:0000717), Increased muscle tone (HP:0001276), Velvety skin (HP:0000977), Hyperextensibility of the finger joints (HP:0001187), Delayed speech and language development (HP:0000750), Toe walking (HP:0040083), Flat feet (HP:0001763), Valgus foot deformity (HP:0008081) | *SLC6A8,* NM_005629.3: c.[224T>C];[=]; p.(Val75Ala); missense, novel; *probably pathogenic;*  *de novo* | Developmental delay, autistic spectrum behavioral changes, carrier females may show neuropsychologic impairment, decreased circumference, myopathic facies, gastrointestinal problems, hyperextensible joints, stub thumb, decreased creatine signal on magnetic resonance spectroscopy^8,9^ |
| Patient 8; 4y; f;  Mental retardation, autosomal dominant 31, MRD31; AD  (MIM 616158) | Intellectual disability (HP:0001249), Self-injurious behavior (HP:0100716), Obesity (HP:0001513), Muscular hypotonia (HP:0001252), Loose skin (HP:0000973), Global developmental delay (HP:0001263), Delayed speech and language development (HP:0000750), Impaired social interactions (HP:0000735), Aggressive behavior (HP:0000718), Decreased fetal movement (HP:0001558), Somnolence (in newborn period) (HP:0001262), Apathy (in newborn period) (HP:0000741), Poor sucking (in newborn period) (HP:0002033), Difficulty in walking (HP:0002355), Hypomimic face (HP:0000338), Broad face (HP:0000283), Blond hair (HP:0002286), Narrow forehead (HP:0000341), Hypertelorism (HP:0000316), Epicanthus (HP:0000286), Thick helix (HP:0000391), Prominent ear lobes (HP:0009748), Upturned nasal tip (HP:0000463), Long philtrum (HP:0000343), Downturned corners of mouth (HP:0002714), Triangular shaped mouth (HP:0000207), Thin upper lip (HP:0000219), Abnormal dermatoglyphics (HP:0007477), Clinodactyly of the 5th finger (HP:0004209), Hyperextensibility of the knee (HP:0010500), Pes planus (HP:0001763), Valgus foot deformity (HP:0008081), Hypoplastic female external genitalia (HP:0012815) | *PURA,* NM_005859.4: c.[3G>A];[=]; p.?;  start loss, novel;  *probably pathogenic*  (SIFT: Deleterious, MutationTaster: disease causing), MAF=0;  *de novo* | Severe delayed psychomotor development, poor/absent speech, seizures, respiratory insufficiency, feeding difficulties, myopathic face with open mouth^10^ |

*The nomenclature of molecular variants follows the Human Genome Variation Society guidelines (HGVS, http://varnomen.hgvs.org/) using human cDNA sequences from RefSeq database

**Molecular variants were assessed by pathogenicity prediction tools: SIFT and MutationTaster software for nucleotide changes localized in coding sequence, and MaxEnt, NNSPLICE or SSF for nucleotide changes identified in intronic sequence. ClinVar database was search for known pathogenic variants (<https://www.ncbi.nlm.nih.gov/clinvar/>). The minor allele frequency (MAF) as recorded in ExAC and GnomAD databases.

Table References

1. Menke LA, study DDD, Gardeitchik T, et al. Further delineation of an entity caused by CREBBP and EP300 mutations but not resembling Rubinstein-Taybi syndrome. *Am J Med Genet A.* 2018;176(4):862-876.

2. Jameel M, Klar J, Tariq M, et al. A novel AP4M1 mutation in autosomal recessive cerebral palsy syndrome and clinical expansion of AP-4 deficiency. *BMC Med Genet.* 2014;15:133.

3. Tuysuz B, Bilguvar K, Kocer N, et al. Autosomal recessive spastic tetraplegia caused by AP4M1 and AP4B1 gene mutation: expansion of the facial and neuroimaging features. *Am J Med Genet A.* 2014;164A(7):1677-1685.

4. Docker D, Schubach M, Menzel M, et al. Further delineation of the SATB2 phenotype. *Eur J Hum Genet.* 2014;22(8):1034-1039.

5. Huisman S, Mulder PA, Redeker E, et al. Phenotypes and genotypes in individuals with SMC1A variants. *Am J Med Genet A.* 2017;173(8):2108-2125.

6. Zarate YA, Smith-Hicks CL, Greene C, et al. Natural history and genotype-phenotype correlations in 72 individuals with SATB2-associated syndrome. *Am J Med Genet A.* 2018;176(4):925-935.

7. Kaiwar C, Zimmermann MT, Ferber MJ, et al. Novel NR2F1 variants likely disrupt DNA binding: molecular modeling in two cases, review of published cases, genotype-phenotype correlation, and phenotypic expansion of the Bosch-Boonstra-Schaaf optic atrophy syndrome. *Cold Spring Harb Mol Case Stud.* 2017;3(6).

8. van de Kamp JM, Mancini GM, Pouwels PJ, et al. Clinical features and X-inactivation in females heterozygous for creatine transporter defect. *Clin Genet.* 2011;79(3):264-272.

9. van de Kamp JM, Betsalel OT, Mercimek-Mahmutoglu S, et al. Phenotype and genotype in 101 males with X-linked creatine transporter deficiency. *J Med Genet.* 2013;50(7):463-472.

10. Hunt D, Leventer RJ, Simons C, et al. Whole exome sequencing in family trios reveals de novo mutations in PURA as a cause of severe neurodevelopmental delay and learning disability. *J Med Genet.* 2014;51(12):806-813.
